# Higher Steroid Production in the Right Adrenal Gland Compared to the Left One in db/db Mice, a Model of Type 2 Diabetic Obesity

**DOI:** 10.3390/ijms251910658

**Published:** 2024-10-03

**Authors:** Rengui Saxu, Qiming Luo, Yong Yang, Harvest F. Gu

**Affiliations:** 1 Laboratory of Molecular Medicine, School of Basic Medicine and Clinical Pharmacy, China Pharmaceutical University, Nanjing 210009, China; 17320056317@163.com; 2 State Key Laboratory of Natural Medicines, School of Traditional Chinese Pharmacy, China Pharmaceutical University, Nanjing 211198, China; luo_qm@163.com; 3 Center for New Drug Safety Evaluation and Research, State Key Laboratory of Natural Medicines, China Pharmaceutical University, Nanjing 211198, China

**Keywords:** adrenal gland, left–right asymmetry, corticosterone, aldosterone, db/db mice

## Abstract

Vertebrates exhibit a left–right asymmetry from the central structures to the peripheral paired endocrine organs. However, the asymmetries in paired endocrine glands and the pathological consequences of such asymmetries remain largely unknown. The adrenal gland constitutes a pair of peripheral end organs in the neuroendocrine system, responsible for producing steroid hormones under stimuli. In the present study, the lateralized asymmetry of left and right adrenal glands in leptin receptor-deficit db/db mice was investigated. First, a morphological and histological examination showed that adrenal mass and adrenal cortex volume in db/db mice were significantly higher than in non-diabetic control mice. Then, adrenal transcriptomic and serum metabolomic analyses were performed. Adrenal steroid profiling showed that the levels of corticosterone and aldosterone in the right adrenal gland of db/db mice were two times higher than in the left one. The expression of multiple genes related to adrenal regeneration and innervation in db/db mice was reduced in contrast to the increased steroid hormone secretion. Furthermore, an examination of morphogens in asymmetric adrenal development revealed a significant differential expression of Shh and its receptor gene Ptch1. In conclusion, the present study has provided evidence that a superior steroidogenesis exists in the right adrenal gland of db/db mice and suggested that Shh signaling may play an important role in asymmetric adrenal responses in type 2 diabetes and its complications.

## 1. Introduction

Adrenal steroid hormones play an important role in regulating many physiological processes, including stress responses, metabolic homeostasis, and sodium reabsorption [1]. These steroid hormones mainly include glucocorticoids (cortisol in humans and corticosterone in rodents) and mineralocorticoids (chiefly aldosterone), which are secreted from two distinct functional zones of the adrenal cortex, the zona fasciculata and the zona glomerulosa [2]. The regulation of adrenocortical development and maintenance includes both hormonal and neural regulatory processes. Hormonal factors mainly include the adrenocorticotropic hormone (ACTH) and angiotensin II (Ang II) via the hypothalamus–pituitary–adrenocortical (HPA) axis and the renin–angiotensin–aldosterone system (RAAS), while the neural control of adrenal function relies on the nerves projecting to the glands [3,4]. Adrenal glands are a pair of peripheral end organs of the neuroendocrine system. The asymmetric neural innervation between the left and right adrenal glands has attracted attention in research. Hala’sz and Szenta’gothai first raised the possibility of asymmetric innervation of the left and right adrenal glands and Gerendai et al. then confirmed that following unilateral adrenalectomy, there was an increase in the protein synthesis activity of hypothalamic ventromedial neurons contralateral to the removed adrenal gland [5,6,7]. Holzwarth and Dallman showed that lesions in the hypothalamus hemi-islands, on the same side as a removed adrenal, might inhibit the neural-driven growth of the adrenal gland [8]. Using a viral trans-neuronal tracing method, Toth et al. revealed the presence of side-specific neurons on either side and demonstrated the dominance of supraspinal innervation in the left adrenal gland over the right [9]. Furthermore, Droste et al. demonstrated that long-term voluntary exercise in mice increased the thickness of the right adrenal cortex compared to the left adrenal cortex, while this effect was abolished by combination with antidepressant therapy [10,11]. Side-specific endocrine signaling disruptions between central structures and peripheral endocrine glands have been linked to neuroendocrine disorders and psychiatric disorders, which show hallmarks of the dysfunction of the HPA axis [12,13]. In the endocrine end organs of the hypothalamus–pituitary axis, such as the testicles and ovaries, similar sided differences were observed. In the testes, left-sided innervation dominance was identified, and higher testosterone concentration was detected in the remaining right testicular vein after unilateral orchidectomy [7,14]. In the ovaries, side-specific innervation was observed, and unequal sex steroid secretion was detected in the left and right ovaries following vasoactive intestinal peptide stimulation [3,15].

Our research group has recently performed transcriptomic analyses and identified miR-466i-4p as an important mediator of asymmetric neural innervation between left and right adrenals in C57BL/6 mice, with higher expression of multiple genes involved in steroidogenesis in the right adrenal compared to the left adrenal [16]. In the present study, we investigated the asymmetric responses between the left and right adrenal glands in db/db (leptin receptor-deficit) mice, a commonly used animal model to mimic human type 2 diabetes (T2D) [17]. In this animal model, the negative feedback regulation of leptin is missing, thus promoting hyperphagia and the subsequent development of obesity [18]. Understanding the pathologic implications of adrenal lateralized asymmetry in db/db mice could provide new insights into the developmental biology of managing metabolic disorders that involve adrenal dysfunction such as T2D and its complications.

## 2. Results

### 2.1. Morphological and Histological Asymmetry

**Figure 1:**
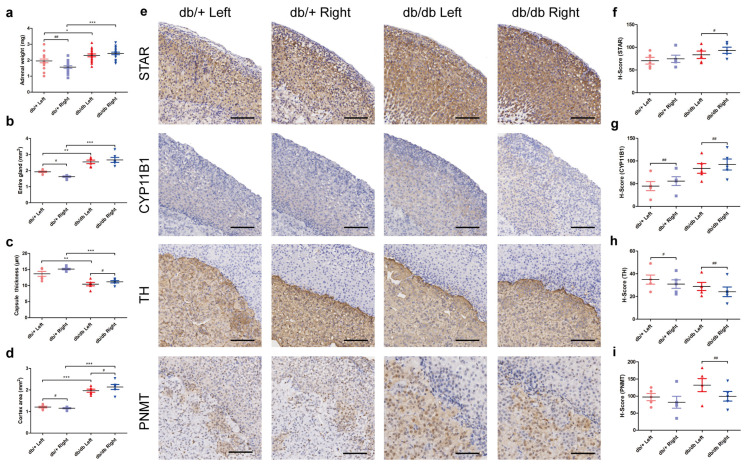
Histological parameters of different adrenal compartments and immunohistochemistry (IHC) in db/+ and db/db mice bilateral adrenals. Adrenal weight (**a**), entire adrenal area (**b**), capsule thickness (**c**), cortex area (**d**) in H&E slides, immunohistochemistry (**e**), and immunostaining histoscore of major adrenal zone markers, including STAR (**f**), CYP11B1 (**g**), TH (**h**), and PNMT (**i**). * *p* < 0.05, ** *p* < 0.01, *** *p* < 0.001, one-way ANOVA followed by Tukey’s test. ^#^ *p* < 0.05, ^##^ *p* < 0.01, paired *t* test or Wilcoxon matched pairs test. The number of animals for histological and IHC analyses were 6 or 5 in each group. The bilateral adrenal samples were used with three slides for each experiment. Scale bars are 100 µm.

At first, an evident morphological difference in the bilateral adrenal glands was observed in both mouse models by using a body field microscope Motic SMZ-168 (Motic Germany GmbH, Wetzlar, Germany). Either in db/+ or db/db mice, the left adrenal gland was typically triangle- or crescent-shaped, whereas the right adrenal gland was characterized as pyramidal or wedge-shaped (Figure S1a). The adrenal weights and histological parameters were then compared. In the db/+ mice, a higher adrenal weight (Figure 1a), adrenal volume (Figure 1b), cortex volume (Figure 1d), and medulla volume (Figure S1e) were detected in the left adrenal gland, whereas the right adrenal gland exhibited a greater cortex/gland ratio and cortex/medulla ratio (Table 1). In db/db mice, the cortex volume and capsule thickness (Figure 1c) of the right adrenal gland were larger, as previously described in other adrenal stress models [10,11]. In a comparative analysis of adrenal glands on the same side in different mouse models, db/db mice exhibited a significantly higher adrenal weight, cortex volume, zG volume (Figure S1c), and zF volume (Figure S1d), but a thinner capsule compared to db/+ mice, suggesting an increased steroid hormone secretion but a reduced adrenal regeneration capacity in db/db mice. The H&E staining images are represented in Figure S1b. Figure S1f depicts the lipid storage in the adrenals of db/+ and db/db mice. Figure S2a–c demonstrates a notable increase in fasting body weight, fasting blood glucose, and fasting serum insulin levels in db/db mice, indicating the successful establishment of a diabetic model.

### 2.2. Comparison of Major Markers of Adrenal Compartments

Immunohistochemistry staining was performed on the adrenal glands of db/+ and db/db mice to compare the different adrenal zones. The STAR and CYP11B1 genes act as markers for the cortex and zF, respectively, and the expression of the TH and PNMT genes help identify the medulla and mature chromaffin cells (Figure 1e). As shown in Figure 1f,g, the immunostaining H-score of the cortex marker STAR and zF marker CYP11B1 was slightly elevated in db/db mice but not statistically significant. When comparing the left and right adrenals, the expression of these cortex markers was higher in the right adrenal in db/db mice. Figure 1h,i show the immunostaining H-score of the medulla marker TH and the adrenalin-producing chromaffin cell marker PNMT. When comparing the left and right adrenals, the expression of TH in left adrenal of db/+ mice was higher, which may be linked to the neural origin of adrenal medulla. In db/db mice, the expression of both TH and PNMT was higher in the left adrenal gland.

### 2.3. DEGs Involved in Adrenal Steroidogenesis

**Figure 2:**
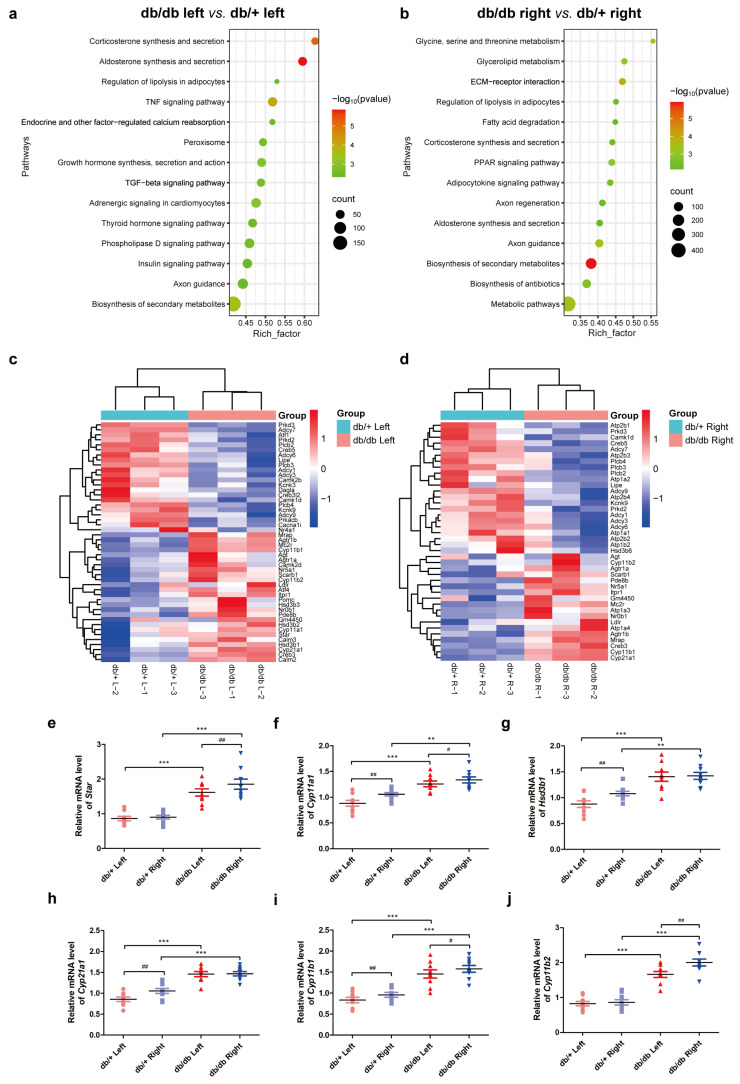
Transcriptomic analysis and validation of DEGs involved in steroid hormone production. Significantly enriched pathways in db/db left vs. db/+ left (**a**) and db/db right vs. db/+ right (**b**), and the heatmaps of DEGs in “Corticosterone synthesis and secretion” and “Aldosterone synthesis and secretion” pathways (**c**,**d**). Relative expression of multiple genes involved in adrenal steroidogenesis, including *Star* (**e**), *Cyp11a1* (**f**), *Hsd3b1* (**g**), *Cyp21a1* (**h**), *Cyp11b1* (**i**), and *Cyp11b2* (**j**). ** *p* < 0.01, *** *p* < 0.001, one-way ANOVA followed by Tukey’s test. ^#^ *p* < 0.05, ^##^ *p* < 0.01, paired *t* test or Wilcoxon matched pairs test.

To elucidate the transcriptomic differences in the adrenals of db/+ and db/db mice, DEGs in the bilateral adrenals were identified in both mouse models. A total of 5686 (db/db left vs. db/+ left) and 5111 (db/db right vs. db/+ right) DEGs were identified comparing the same side of db/db and db/+ mice, and a total of 1577 (db/db right vs. db/db left) and 2566 (db/+ right vs. db/+ left) DEGs were identified when comparing the contralateral adrenals in db/db mice and db/+ mice, respectively, with a differential expression threshold of fold change (FC) exceeding 1.2 and a false discovery rate (FDR) below 0.001. Then, the enrichment of the KEGG pathway was conducted by comparing db/db left vs. db/+ left and db/db right vs. db/+ right, resulting in a significant enrichment of the “Corticosterone synthesis and secretion” and “Aldosterone synthesis and secretion” pathways (Figure 2a,b). DEGs in these pathways were represented as a heatmap (Figure 2c,d). A Venn diagram indicated the overlapping DEGs present in the left–right differences in both mouse models and a comparison of left–right DEGs in db/db mice versus left–right DEGs in db/+ mice revealed 467 common genes. Furthermore, pathway enrichment of these common DEGs identified a significant enrichment of pathways responsible for steroid hormone secretion, including “Corticosterone synthesis and secretion” and “Aldosterone synthesis and secretion”, as well as pathways related to protein synthesis and energy metabolism, including “Ribosome”, “Thermogenesis”, and “Oxidative phosphorylation”, which suggested lateralized asymmetries in steroidogenesis and energy metabolism in bilateral adrenals in both mouse models (Table 2). Then, various key genes involved in steroid hormone production including *Star*, *Cyp11a1*, *Hsd3b1*, *Cyp21a1*, *Cyp11b1*, and *Cyp11b2* were validated by RT-qPCR (Figure 2e–j).

### 2.4. Serum Metabolomics and Adrenal Steroid Profiling

**Figure 3:**
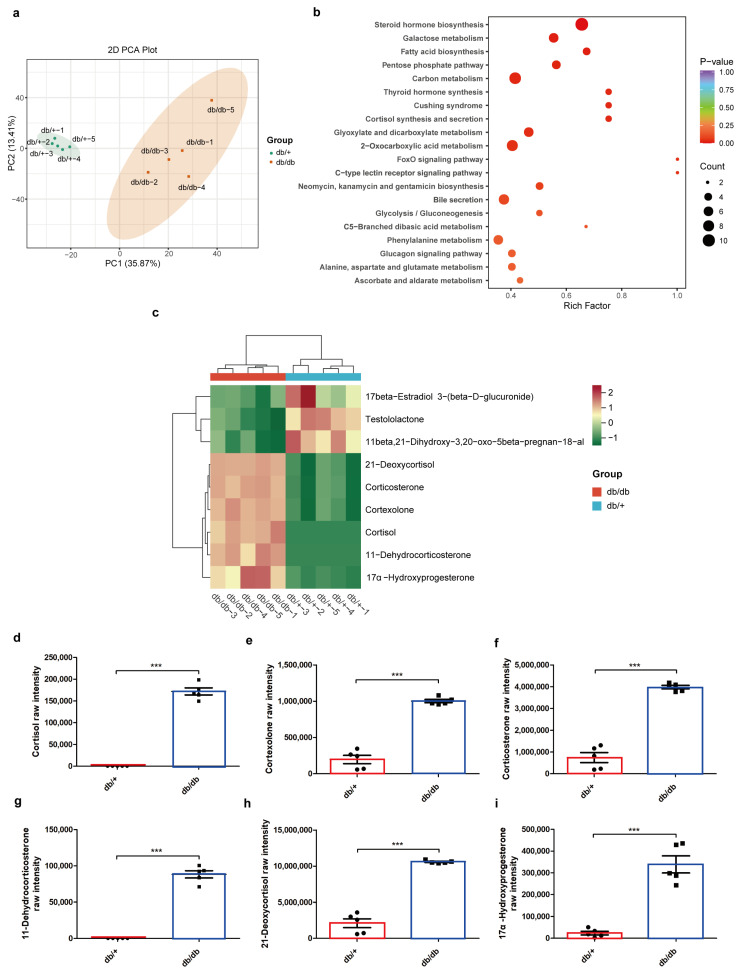
Serum metabolomics in db/+ and db/db mice. PCA plot (**a**), KEGG pathway enrichment analysis (**b**), differential metabolites in the significantly enriched pathway “Steroid hormone production” (**c**), and the raw intensity of significantly unregulated metabolites in this pathway, including cortisol (**d**), cortexolone (**e**), corticosterone (**f**), 11-dehydrocorticosterone (**g**), 21-deoxycortisol (**h**), and 17α-hydroxyprogesterone (**i**). The red boxes represent values in db/+ mice, and the blue box represent values in db/db mice. *** *p* < 0.001, unpaired *t* test with Welch’s correction.

**Figure 4:**
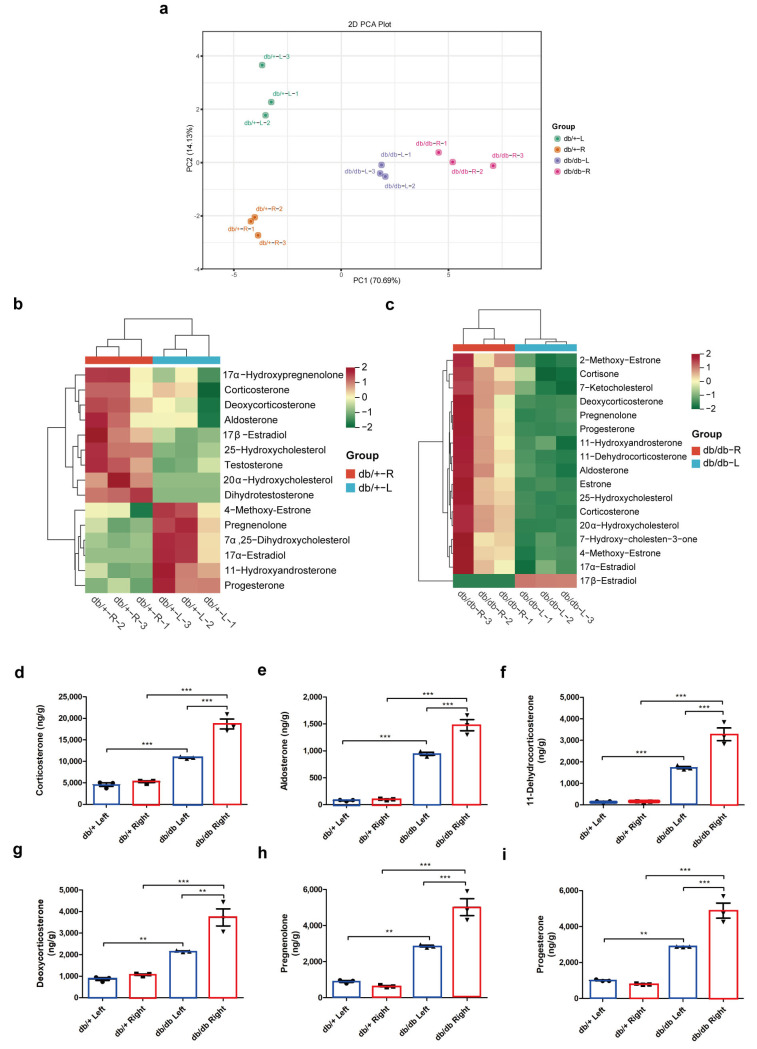
Adrenal steroid profiling. PCA plot (**a**) and heatmaps (**b**,**c**) of differential steroid metabolites in db/+ and db/db mice contralateral adrenals. Significant differential steroid metabolites including corticosterone (**d**), aldosterone (**e**), 11-dehydrocorticosterone (**f**), deoxycorticosterone (**g**), pregnenolone (**h**), and progesterone (**i**). The blue boxes represent the left adrenals, and the red boxes represent the right adrenals. ** *p* < 0.01, *** *p* < 0.001, one-way ANOVA followed by Tukey’s test.

A total of 2025 distinct annotated metabolites were evaluated in the serum of db/+ and db/db mice, and 389 differential metabolites were identified, including 55 small peptides, 49 organic acids and their derivatives, 45 heterocyclic compounds, 39 benzene and substituted derivatives, 22 amino acid derivatives, and 14 hormone and hormone-related compounds (Figure S3a). A PCA plot (Figure 3a) and OPLS-DA (Figure S3b) revealed significant differences between the distributions of samples, suggesting a significant difference in serum metabolites in db/+ and db/db mice, and the most significant pathway, “Steroid hormone biosynthesis”, was identified by pathway enrichment with differential metabolites (Figure 3b). The significantly increased steroid metabolites in this pathway include 11-dehydrocorticosterone, 17α-hydroxyprogesterone, 21-deoxycortisol, cortexolone, corticosterone, and cortisol, which indicate higher steroid hormone levels in db/db mice serum (Figure 3c). Significantly higher raw intensities of these steroids in the serum of db/db mice were shown in Figure 3d–i. Then, a PCA plot of the differential metabolites in the bilateral adrenal steroid profiles of db/+ and db/db mice showed significant differences in the adrenal steroid profiles of db/+ left, db/+ right, db/db left, and db/db right adrenals (Figure 4a). Furthermore, the abundances of metabolites with significantly different values compared to the contralateral adrenal gland in db/+ and db/db mice were shown in heatmaps (Figure 4b,c). The heatmap of adrenal steroid profiles comparing the same side of adrenal glands in db/db vs. db/+ mice is presented in Figure S4a,b. Then, the levels of major steroids including corticosterone, aldosterone, 11-dehydrocorticosterone, deoxycorticosterone, pregnenolone, and progesterone were validated (Figure 4d–i). In db/db mice, an almost two-fold increase in all of these steroids in the right adrenal over the left adrenal was indicated. It demonstrated a significantly potent capacity of the right adrenal gland in steroid production in metabolic disorders.

### 2.5. DEGs Involved in Adrenal Stem Cell Maintenance and Glucose Homeostasis

**Figure 5:**
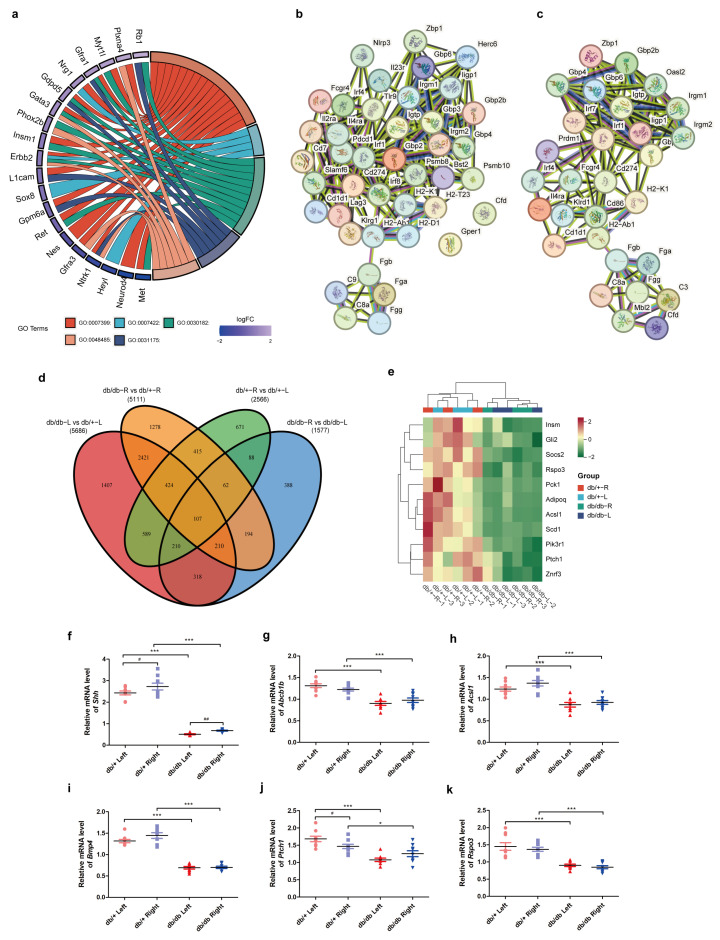
DEGs involved in immune function and adrenal regeneration in the bilateral adrenal glands of db/db mice. Chord plot containing GO terms in adrenal regeneration and innervation (**a**). PPI network involved in immune system process in db/db mice left and right adrenals (**b**,**c**). Venn diagram of common DEGs between groups (**d**). Heatmap of DEGs relating to adrenal dysfunction and type 2 diabetes (**e**) and validation of key genes in the development of diabetes in db/db mice adrenals, including *Shh* (**f**), *Abcb1b* (**g**), *Acsl1* (**h**), *Bmp4* (**i**), *Ptch1* (**j**), and *Rspo3* (**k**). * *p* < 0.05, *** *p* < 0.001, one-way ANOVA followed by Tukey’s test. ^#^ *p* < 0.05, ^##^ *p* < 0.01, paired *t* test or Wilcoxon matched pairs test.

By GO enrichment of the same side of db/+ and db/db, GO:0007399, nervous system development was significantly enriched, and significantly enriched four lower levels of GO terms related to adrenal nervous system development including GO:0007422 for peripheral nervous system development, GO:0030182 for neuron differentiation, GO:0031175 for neuron projection development, and GO:0048485 for sympathetic nervous system development were collected. Then, a chord graph with 20 common DEGs between these GO terms, including *Met*, *Neurod4*, *Heyl*, *Ntrk1*, *Gfra3*, *Nes*, *Ret*, *Gpm6a*, *Sox8*, *L1cam*, *Erbb2*, *Insm1*, *Phox2b*, *Gata3*, *Gdpd5*, *Nrg1*, *Gfra1*, *Myt1l*, *Plxna4*, and *Rb1* was presented (Figure 5a). The expression of these genes in the adrenals of db/db mice was significantly lower than that of db/+. *Shh* is a laterality gene that plays an important role in the embryo left–right patterning and adult adrenal stem cell maintenance [19,20]. In the present study, the expression of *Shh* was significantly reduced in db/db mice, as was the expression of its ligand-binding receptor gene *Ptch1*. In addition, the expression of *Shh* in the right adrenal was significantly higher than the left adrenal in both db/+ and db/db mice (Figure 5f). Furthermore, PPI networks were constructed, and the major networks responsible for “Immune system process” and “Complement and coagulation cascades” were identified by comparing the same side of the db/db and db/+ mouse adrenals (Figure 5b,c). In addition, several common DEGs derived from the Venn diagram have been linked to adrenal disorders and the development of type 2 diabetes (Figure 5d), according to the T2D Knowledge Portal (https://t2d.hugeamp.org/, accessed on 20 December 2023). These genes included *Acsl1*, *Adipoq*, *Pck1*, *Scd1*, *Gli2*, *Znrf3*, *Rspo3*, *Socs2*, *Insm1*, *Pik3r1*, and *Ptch1* (Figure 5e). The expression of several of these genes was validated by RT-qPCR, and significantly reduced expression levels of these genes were observed in db/db mice (Figure 5g–k).

## 3. Discussion

In the present study, the morphological, histological, transcriptomic, and serum metabolomics analyses, as well as adrenal steroid profiling were performed on the adrenal glands of db/+ and db/db mice with a special focus on left–right asymmetry. First, a significantly higher level of steroid hormones was detected in db/db mice serum compared to non-diabetic db/+ mice. More importantly, the analysis of adrenal steroid metabolites revealed that the steroid production in the right adrenal was even double that in the left adrenal, suggesting that the right adrenal gland was dominant in steroid production in db/db mice. In the db/+ mice, the weight and size of the left adrenal was larger, while this feature was reversed in the db/db mice, which has also been reported in several stress animal models [10,11] with a more significant enlargement of the right adrenal cortex suggested under stress [21]. In db/+ mice, despite the right adrenal gland exhibiting less adrenal weight and volume, there was a greater cortex/gland ratio and cortex/medulla ratio compared to the left adrenal gland. In db/db mice, the capsule thickness and cortex volume of the right adrenal was higher. Among the steroidogenesis-related genes, the expression of *Star* in db/+ mice was not as statistically high in the right adrenal as seen in C57BL/6 mice [16]. In addition to the difference in mouse strain, low gene expression fold change in the Star gene between the left and right adrenal may lead to this variation.

Furthermore, investigations of morphogens involved in the development of the adrenal glands identified Shh as a key regulator in the lateralized asymmetries of left and right adrenal glands, both in terms of morphology and function. It has been reported that the mice with Shh^fl/fl;SF−1/Cre+^ only exhibited a detectable left adrenal gland, with the right adrenal gland being unobservable [22]. Another study has demonstrated that the targeted removal of the Shh gene in steroidogenic cells in mouse adrenals resulted in distinct effects on the left and right adrenal glands [23]. Shh signaling is a crucial morphogen in vertebrates that controls the development of the central nervous system and limbs in a tightly regulated dose- and time-dependent manner [19,24]. In the adrenal gland, Shh also plays an important role in adrenal development and adrenocortical regeneration in adults [20,25]. In the present study, Shh was significantly more expressed in the right adrenal compared to the left in both db/db and db/+ mice. In addition, the interaction of Shh signaling with Ptch1, a ligand-binding inhibitor of Shh, was observed in this study, as previously suggested by [22,24,26]. Their gene expression was validated by RT-qPCR in the present study, while more studies are needed to clarify the exact Shh-related gene cascade involved in the lateralized asymmetry in adrenal glands.

In addition to the increased steroid hormones, the levels of several precursors of steroid hormones were reduced in the adrenal glands of db/db mice compared to db/+ mice. Desmosterol is the intermediate precursor of cholesterol synthesis, and 17alpha-hydroxypregnenolone is a precursor of progesterone [27,28]. Excess production of steroid hormones may exhaust these steroid hormone precursors, thus leading to the reduction in these precursors in db/db mice adrenals. Testosterone and dihydrotestosterone are major androgens, which were reduced in the adrenal glands of male db/db mice, while low circulating androgen levels were associated with obesity in males characterized as increased fat mass and reduced lean mass [29].

Adrenal innervation plays an important role in many aspects of the adrenal gland, such as compensatory adrenal growth, the diurnal rhythm of plasma corticosterone, and adrenal regeneration [8,30,31]. In the present study, the expression of several genes involved in adrenal innervation and adrenal development were significantly reduced in db/db mice. In addition, reduced immune function was also suggested in db/db mice by PPI network establishment. Furthermore, the pathways responsible for steroidogenesis, including “Corticosterone synthesis and secretion” and “Aldosterone synthesis and secretion”, were significantly enriched by constructing the Venn diagram using the common left vs. right adrenal DEGs in both db/db and db/+ mice, consistent with the right-sided dominance of steroid hormone secretion. In addition, the pathways “Ribosome”, “Thermogenesis”, and “Oxidative phosphorylation” were enriched, and genes in these pathways were more expressed in the left adrenal. This is consistent with the higher organ weight and more abundant innervation of the left adrenal in db/+ mice [9,10,11]. In db/db mice, the right adrenal weight was higher, possibly due to a significant enlargement of the right adrenal cortex as previously described. It has been suggested that these sided differences in organ weight, side-specific innervation, and distinct energy metabolism may be related to the different incidence of cancer in the left and right adrenal glands [32,33,34], as observed in other paired organs such as the breast, kidney, and ovaries [35,36,37]. In addition, the morphology, vascular supply, and venous drainage of the left and right adrenals are also different, all of which in combination may result in completely different molecular actions in the left and right adrenals, further resulting in diverse functional activity [7,33]. The adrenal gland is a sexually dimorphic endocrine organ. In addition to the time-dependent adrenal development from weaning to adulthood, the sex difference affects adrenal development primarily in the sex hormone precursor secreted from the zona reticularis (X-zone in mice, which regresses at sexual maturity in males and during the first pregnancy in females). In the present study, we have focused on the study of the left–right asymmetry of adrenal glands in male db/db mice. Further investigation of the left–right asymmetry of adrenal glands in females has been taken into our consideration.

In conclusion, the present study found that db/db mice exhibit a significant right adrenal dominance in corticosterone and aldosterone secretion, in which Shh signaling may play an important role. This evidence may be beneficial for the management of type 2 diabetes and various endocrine disorders with adrenal dysfunction.

## 4. Materials and Methods

### 4.1. Morphological and Histological Analysis

Male db/db mice (homozygous C57BLKS-Leprdb) at the age of 11 weeks and either sex- or age-matched control db/m mice (C57BLKS-Leprdb/+) (hereafter referred to as ‘‘db/+’’) were obtained from the Model Animal Research Center of Nanjing University (Nanjing, China). The animals were bred in temperature-controlled (24 ± 2 °C) and constant humidity (55 ± 10%) conditions on a 12 h light/dark cycle (lights on at 7:00 a.m.), offering free access to regular food and water for one week at the Animal Experimental Center of China Pharmaceutical University (Nanjing, China). The overnight fasting animals were anesthetized with 4% isoflurane (RWD Life Science, Shenzhen, China) in an induction chamber and then blood samples were collected by eyeball extirpating. Anesthesia was maintained with 1.5–2% isoflurane delivered via a facemask and cardiac perfusion was performed through the left cardiac ventricle with 0.9% sodium chloride solution to remove the peripheral blood in the adrenal vessels. Then, the mice were euthanized with cervical dislocation and bilateral adrenals were collected. The fasting serum insulin levels were detected with the Mouse insulin ELISA kit (no. E-EL-M1382c, Elabscience Biotechnology Co., Ltd., Wuhan, China). All experimental procedures were conducted between 08:00 and 10:00 h. Animal experiments in this study complied with the China Pharmaceutical University Guide for Laboratory Animals and the Declaration of Helsinki. The Institutional Animal Care and Concern Committee at China Pharmaceutical University approved all experimental protocols.

### 4.2. H&E and Oil Red O Staining

The adrenal glands (*n* = 6) were fixed in 4% paraformaldehyde for 24 h at 4 °C before being embedded in paraffin, dehydrated in alcohol, and washed in xylene. A size of 5 μm thick cross-sections was cut from the blocks and stained with hematoxylin and eosin. Digital images were obtained using a whole slide pathology scanner Leica Aperio VESA8 (Leica Biosystems, Wetzlar, Germany). The program enables the merging of digital pictures taken during the scanning of a slide show into a harmoniously united composite image. The areas of the entire gland, capsule, medulla, and zona glomerulosa (zG) were quantified using the Image J Fiji software (version 1.53q, Wayne Resband and contributors, National Institutes of Health, Rockville, MD, USA) on the three largest equatorial sections of each adrenal gland [38,39]. The adrenal cortex area was determined by subtracting the capsule and medullary areas from the entire gland. To calculate the zona fasciculata (zF) area, the zG was subtracted from the cortex, as there is no distinct zona reticularis in adult male mice [40]. By analyzing the volume of the various compartments, the ratios of the capsule, cortex, and medulla within the entire gland were quantified, as well as the ratios of zG and zF within the cortex.

Cholesterol serves as a precursor to steroid hormones, and excess cholesterol is stored in lipid droplets as cholesterol esters [41]. To evaluate the intracellular storage of cholesterol, oil red O staining of lipid droplets was performed in db/db and db/+ mice bilateral adrenals (*n* = 4). A series of cryo-sections of the bilateral adrenals were cut and then thaw-mounted on a pre-coated slide. The 5 μm sections were fixed in 4% paraformaldehyde for 1 min and then rinsed in 60% isopropyl alcohol for 3 min and stained with the oil red O staining solution (Sigma, Darmstadt, Germany). Hematoxylin was used for counterstaining. The mosaic images were captured with the Leica HS6 digital whole slide pathology scanner (Leica Microsystems, Wetzlar, Germany) and the area of lipid droplets [pixels] was measured using Image J Fiji software. The adrenal medulla, which is distinguishable by eye, was excluded from the analysis, as steroid hormones are exclusively secreted from the adrenal cortex.

### 4.3. Immunohistochemistry

The adrenal slides (*n* = 5) were embedded in paraffin and cut into sections, then treated with 4% paraformaldehyde. For dehydration, xylene was used, followed by alcohol. After antigen extraction, slides were treated with the primary antibodies. The sections were then treated with the secondary antibody and DAB detection equipment, and counterstained with hematoxylin and dehydrated. The digital images were acquired by a scanner Leica Aperio VESA8 and then analyzed using the whole slide image analysis platform HALO software, version 3.4.2986 (Indica Labs, Albuquerque, NM, USA). The histoscore (H-score), ranging from 0 to 300, was determined by semi-quantitatively evaluating both the intensity of the staining and the proportion of positive cells. The antibodies were purchased from Proteintech Group (Rosemont, IL, USA), Santa Cruz Biotechnology (Dallas, TX, USA), Abcam (Waltham, MA, USA), or GeneTex (Irvine, CA, USA). The antibodies and their respective dilutions employed in the study are as listed as follows: rabbit anti-STAR antibody (1:200, 12225-1-AP, Proteintech), mouse anti-CYP11B1 antibody (1:50, sc-377401, Santa Cruz), rabbit anti-TH antibody (1:500, ab137869, Abcam), and rabbit anti-PNMT antibody (1:400, GTX114098, GeneTex).

### 4.4. Transcriptomic Analysis

Total RNAs were extracted from adrenal tissue using TRIZOL Reagent (Invitrogen, Carlsbad, CA, USA) in accordance with the manufacturer’s instructions. RNA sample integrity was evaluated using a NanoPhotometer^®^ spectrophotometer (IMPLEN, Munich, Germany) and the concentration of RNA was determined using a Qubit^®^ 2.0 Fluorometer (Life Technologies, Carlsbad CA, USA). The RNA integrity number (RIN) was ascertained using the Agilent Bioanalyzer 2100 system (Agilent Technologies, Palo Alto, CA USA). Sequencing libraries for each RNA sample were constructed using a TruseqTM RNA Sample Prep Kit (Illumina, San Diego, CA, USA) according to the manufacturer’s protocol. Subsequent sequencing was performed on a Novaseq 6000 platform (Illumina, San Diego, CA, USA) with paired-end 150 bp reads, and the clean data were used for downstream analysis. The expression level of each gene was measured using the transcript per million reads (TPM) method, and R statistical package EdgeR was used for differential expression analysis. Then, the differentially expressed genes (DEGs) were examined and explained using an online data analysis and visualization platform (http://www.bioinformatics.com.cn/, accessed on 4 May 2022). Gene ontology (GO) analysis and the Kyoto Encyclopedia of Genes and Genomes (KEGG) pathway analysis were performed using Goatools website (https://github.com/tanghaibao/GOatools, accessed on 4 May 2022) and the KOBAS 3.0 online tool (http://bioinfo.org/kobas, accessed on 4 May 2022) [42,43]; a *p*-value < 0.05 was considered to indicate significantly enriched pathways in the present study. The STRING (Search Tool for the Retrieval of Interacting Genes, accessed on 4 May 2022) database was used to construct the protein–protein interaction (PPI) network.

### 4.5. RT-qPCR Validation

Expressions of various key genes involved in adrenal steroidogenesis, including mRNA levels of *Star*, *Cyp11a1*, *Hsd3b1*, *Cyp21a1*, *Cyp11b1*, and *Cyp11b2*, and the genes involved in adrenal stem cell maintenance, including *Shh*, *Abcb1b*, *Acsl1*, *Bmp4*, *Ptch1*, and *Rspo3*, were analyzed using RT-qPCR. The specificity and efficiency of the pairs of primers used for the PCR amplification were checked by the melting curve analysis, and relative gene expressions were calculated using the 2^−ΔΔCt^ method with *Actb* as the endogenous control. The sequence of primers is listed in Supplementary Table S1.

### 4.6. Serum Metabolomics

Fifty μL of serum sample and 300 μL of extraction solution (ACN: methanol = 1:4, *v*/*v*) containing internal standards were vortexed and then centrifuged, and 180 μL aliquots of supernatant were transferred for LC-MS analysis. The sample extracts were analyzed using an LC-ESI-MS/MS system3 (UPLC, ExionLC AD; MS, QTRAP^®^ System, Sciex, Framingham, MA USA). The analytical conditions were as follows: UPLC: column, water ACQUITY UPLC HSS T3 C18 (1.8 μm, 2.1 mm × 100 mm); column temperature, 40 °C; flow rate, 0.4 mL/min; injection volume, 2 μL or 5 μL; solvent system, water (0.1% formic acid)/acetonitrile (0.1% formic acid); and gradient program, 95:5 *v*/*v* at 0 min, 10:90 *v*/*v* at 10.0 min, 10:90 *v*/*v* at 11.0 min, 95:5 *v*/*v* at 11.1 min, and 95:5 *v*/*v* at 14.0 min. LIT and triple quadrupole (QQQ) scans were obtained using a triple quadrupole–linear ion trap mass spectrometer (QTRAP), QTRAP^®^ LC-MS/MS System, equipped with an ESI Turbo Ion-Spray interface (Sciex, Framingham, MA USA), working in positive and negative ion mode and controlled by Analyst 1.6.3 software (Sciex). Multiquant 3.0.3 software (Sciex) was employed for the quantification of the metabolites and raw peak intensities were used to quantify each metabolite.

Unsupervised principal component analysis (PCA) was conducted using the statistical function prompt in R (www.r-project.org, accessed on 5 September 2022). The data were normalized by unit variance before performing unsupervised PCA. Differential metabolites between the two groups were identified based on the variable importance in projection (VIP, VIP ≥ 1) and the absolute value of log2 fold change (|Log2 FC| ≥ 1.0). VIP values were obtained from the OPLS-DA (orthogonal partial least squares discriminant analysis) results, which include both score and permutation plots generated using the R package MetaboAnalystR [44,45]. Prior to OPLS-DA, the data underwent log transformation (log2) and mean centering. The identified metabolites were labeled using the KEGG compound database (http://www.kegg.jp/kegg/compound/, accessed on 5 September 2022), and these annotated metabolites were subsequently linked to the KEGG pathway database (http://www.kegg.jp/kegg/pathway.html, accessed on 5 September 2022). Pathways containing metabolites that were significantly regulated were then analyzed using metabolite sets enrichment analysis (MSEA), and their significance was assessed based on the *p*-values obtained from a hypergeometric test.

### 4.7. Adrenal Steroid Profiling

Each set of 10 left or right adrenal glands was combined into a single sample and vortexed with 400 μL of methanol, and then centrifuged and redissolved with 100 μL of methanol. The 80 μL of supernatant was transferred for further LC-MS analysis. The sample extracts were analyzed using AB 6500+ QTRAP^®^ LC-MS/MS System, operating in the multiple reaction monitoring modes. The analytical conditions were as follows: HPLC: column, Phenomenex Kinetex C18 (1.7 µm, 100 mm × 2.1 mm i.d.) (Phenomenex, Torrance, CA 90501-1430 USA); solvent system, 30% acetonitrile/water with 0.04% acetic acid (A) and 50% acetonitrile/isopropanol with 0.04% acetic acid (B); the gradient was started at 5% B (0–1.0 min), increased to 90% B (1.0–10 min), maintained at 90% B (10–12.5 min), and finally ramped back to 5% B (12.6–15 min); flow rate, 0.35 mL/min; temperature, 40 °C; and injection volume, 5 μL. Analyst 1.6.3 software (Sciex, Framingham, MA USA) was utilized for data acquisitions, and Multiquant 3.0.3 software (Sciex, Framingham, MA USA) was employed for the quantification of steroid metabolites. Significantly regulated steroid metabolites between groups were determined by absolute Log2FC. Adrenal steroid profiling provides the absolute quantitation of 43 steroid metabolites encompassing the precursors, intermediates, and final steroid hormones in the adrenal glands.

### 4.8. Statistical Analysis

The experimental data were expressed as the mean ± SEM. The Kolmogorov–Smirnov test was used to evaluate the normality of all data sets. For the two-group analysis, which included the fasting body weight, fasting blood glucose, and fasting serum insulin levels of db/db and db/+ mice, the Mann–Whitney *U* test or the unpaired *t* test with Welch’s correction were used. For multiple group comparisons, a one-way ANOVA followed by Tukey’s multiple comparisons test was performed. When comparing left and right adrenals from the same animals, a paired *t* test or the Wilcoxon matched pairs test was performed. All statistical analysis was performed using GraphPad Prism 5 software, and *p* < 0.05 was considered as statistically significant.

## Figures and Tables

**Table 1:** Selected morphometric parameters in adrenal glands of db/+ and db/db mice.

Ratio	db/+ Left	db/+ Right	db/db Left	db/db Right
Cortex/gland	0.628 ± 0.039	0.714 ± 0.060 ^a##^	0.776 ± 0.028 ^b^***	0.832 ± 0.060 ^b^**^,c#^
zG/cortex	0.221 ± 0.030	0.212 ± 0.031	0.294 ± 0.077	0.298 ± 0.071
zF/cortex	0.779 ± 0.030	0.788 ± 0.031	0.706 ± 0.077	0.702 ± 0.071
Medulla/gland	0.349 ± 0.055 ^a##,b^***	0.261 ± 0.047	0.206 ± 0.028 ^c#^	0.146 ± 0.064
Cortex/medulla	1.832 ± 0.313	2.822 ± 0.594 ^a##^	3.843 ± 0.783	7.255 ± 4.504 ^b^**^,c#^

Data were means ± SEM. Number of mice is 6. ^a^ Significantly higher value compared to the contralateral adrenals in db/+ mice (^##^ *p* < 0.01 left vs. right, paired *t* test). ^b^ Significantly higher value compared the same-side adrenals of db/db mice to the db/+ (** *p* < 0.01, *** *p* < 0.001 db/+ vs. db/db, Tukey’s multiple comparisons test). ^c^ Significantly higher value compared to the contralateral adrenals in db/db mice (^#^ *p* < 0.05 left vs. right, paired *t* test or Wilcoxon matched pairs test).

**Table 2:** Pathway enrichment of common DEGs comparing the contralateral adrenals in db/db and db/+ mice.

Pathway	Level 1	Level 2	ID	DEG Number	Total Number	*p*-Value	fdr
Ribosome	Genetic information	Translation	ko03010	21	247	3.45 × 10^−9^	4.27 × 10^−7^
Thermogenesis	Organismal systems	Environmental adaptation	ko04714	15	232	8.58 × 10^−4^	0.0423
Corticosterone synthesis and secretion	Organismal systems	Endocrine system	ko04927	8	59	0.0013	0.0445
Non-alcoholic fatty liver disease	Organismal systems	Endocrine and metabolic disease	ko04932	11	128	0.0031	0.0961
Oxidative phosphorylation	Metabolism	Energy metabolism	ko00190	10	141	0.0038	0.1051
Circadian entrainment	Organismal systems	Environmental adaptation	ko04713	8	72	0.0072	0.1774
Neutrophil extracellular trap formation	Organismal systems	Immune system	ko04613	12	88	0.0084	0.1829
Aldosterone synthesis and secretion	Organismal systems	Endocrine system	ko04925	8	84	0.0088	0.1829
Cushing syndrome	Human diseases	Endocrine and metabolic disease	ko04934	10	122	0.0122	0.2320
Transcriptional misregulation in cancer	Human diseases	Cancer: overview	ko05202	12	161	0.0141	0.2484

## Data Availability

All data and material are available from the corresponding author upon reasonable request. RNA sequencing data related to this study can be accessed in the NCBI Sequence Read Archive database with Bioproject ID PRJNA1079824 (https://www.ncbi.nlm.nih.gov/sra/PRJNA1079824, accessed on 23 February 2024).

## Supplementary Materials

The following supporting information can be downloaded at: https://www.mdpi.com/article/10.3390/ijms251910658/s1.
