# Supplementary material for: Higher Steroid Production in the Right Adrenal Gland Compared to the Left One in db/db Mice, a Model of Type 2 Diabetic Obesity
Source: Int J Mol Sci. 2024 Oct 3;25(19):10658. doi: 10.3390/ijms251910658 (PMC11477137; doi:10.3390/ijms251910658)
Supplement: Supplementary file 1 [file ijms-25-10658-s001.zip › ijms-3174730-supplementary.pdf]

Supplementary Materials for

Higher Steroid Production in the Right Adrenal Gland Compared to the Left One in db/db Mice, a Model of Type 2 Diabetic Obesity

Rengui Saxu, Qiming Luo, Yong Yang, Harvest F. Gu

* Correspondence: Harvest F. Gu: feng.gu@cpu.edu.cn Yong Yang: yy@cpu.edu.cn

Supplementary Figures and captions

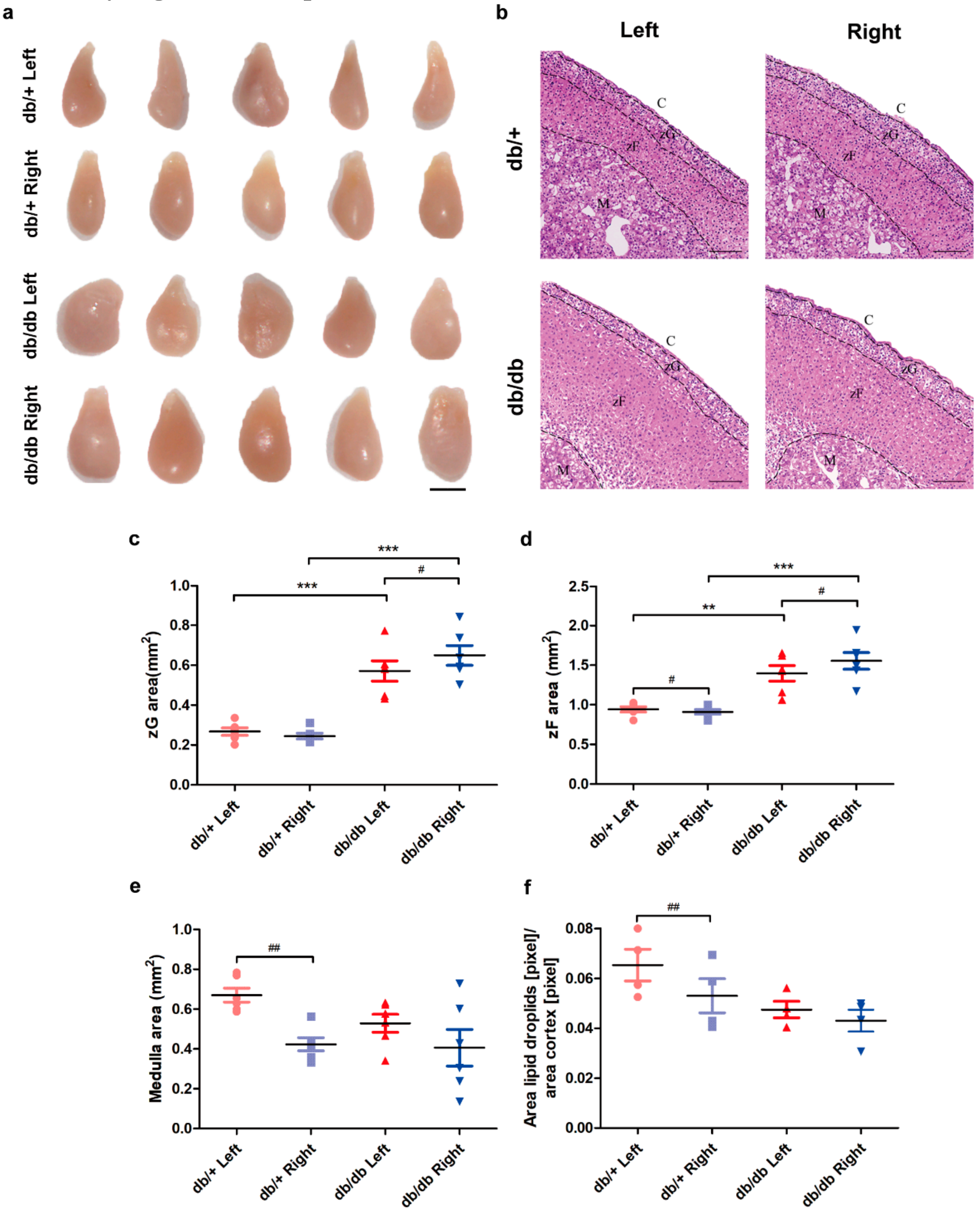

Figure S**1.** Morphology (**a**), histology (**b**), zG area (**c**), zF area (**d**) and medulla area in H&E staining (**e**) and lipid storage analysis (**f**) in db/+ and db/db mice adrenal glands. Scale bars are 0.5 mm for morphological images and 100 μ m for H&E staining. $^{**}p<0.01$, $^{***}p<0.001$, one-way ANOVA followed by Tukey's test. $^{\#}p<0.05$, $^{\#\#}p<0.01$, paired t test.

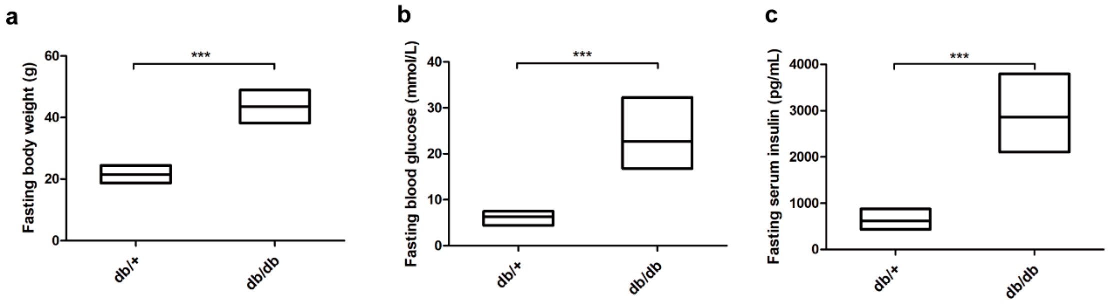

Figure S2. Box plot showing the quartile distribution of fasting body weight (**a**), fasting blood glucose (**b**) and fasting serum insulin (**c**) level of db/+ and db/db mice. $^{***}p<0.001$, Mann–Whitney *U* test was used for statistical analysis of fasting body weight, and unpaired t test with Welch's correction were used for statistical analysis of fasting blood glucose and fasting serum insulin after testing for normality with the Kolmogorov - Smirnov test.

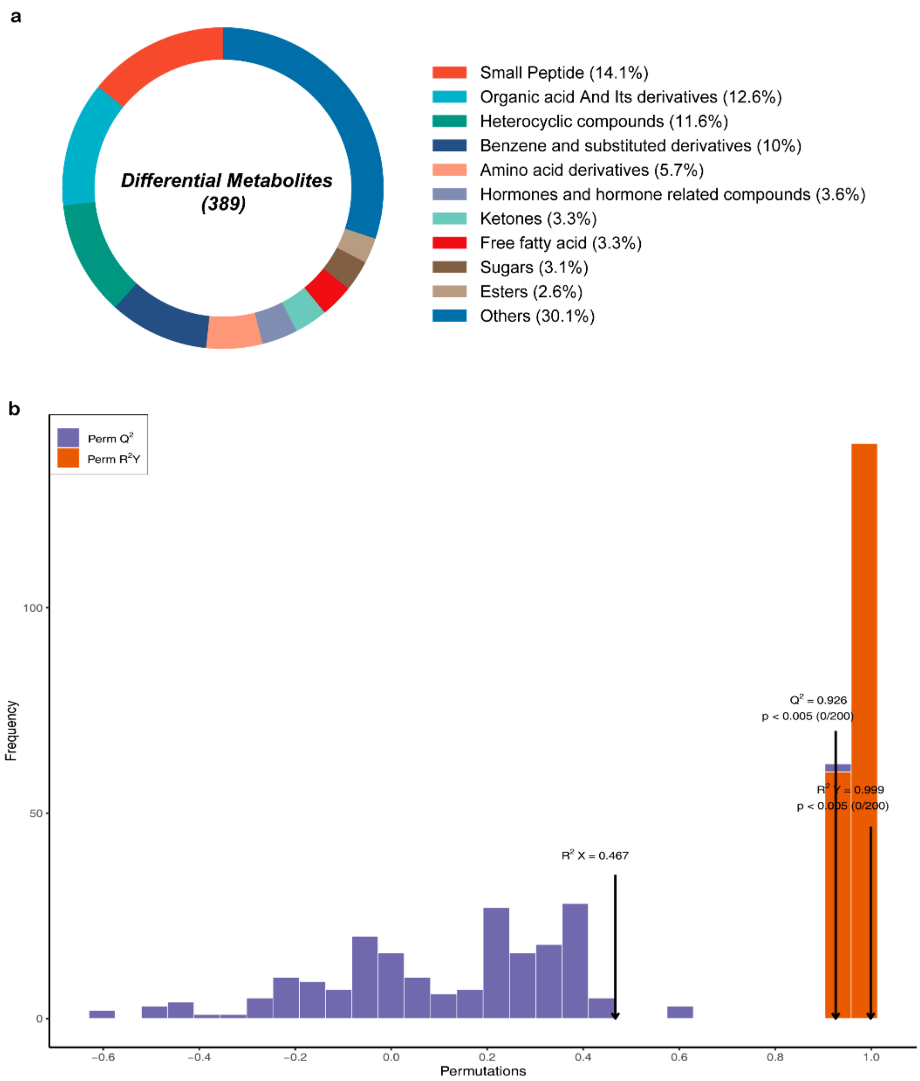

Figure S3. Donut chart showing the differential metabolites in db/+ and db/db mice serum metabolomics (**a**), and the establishment of OPLS-DA model of serum metabolomics (**b**). The OPLS-DA model had good prediction ability, permutation test showed that R^2X (cum) =0.467, R^2Y (cum) = 0.999, Q^2 (cum) = 0.926.

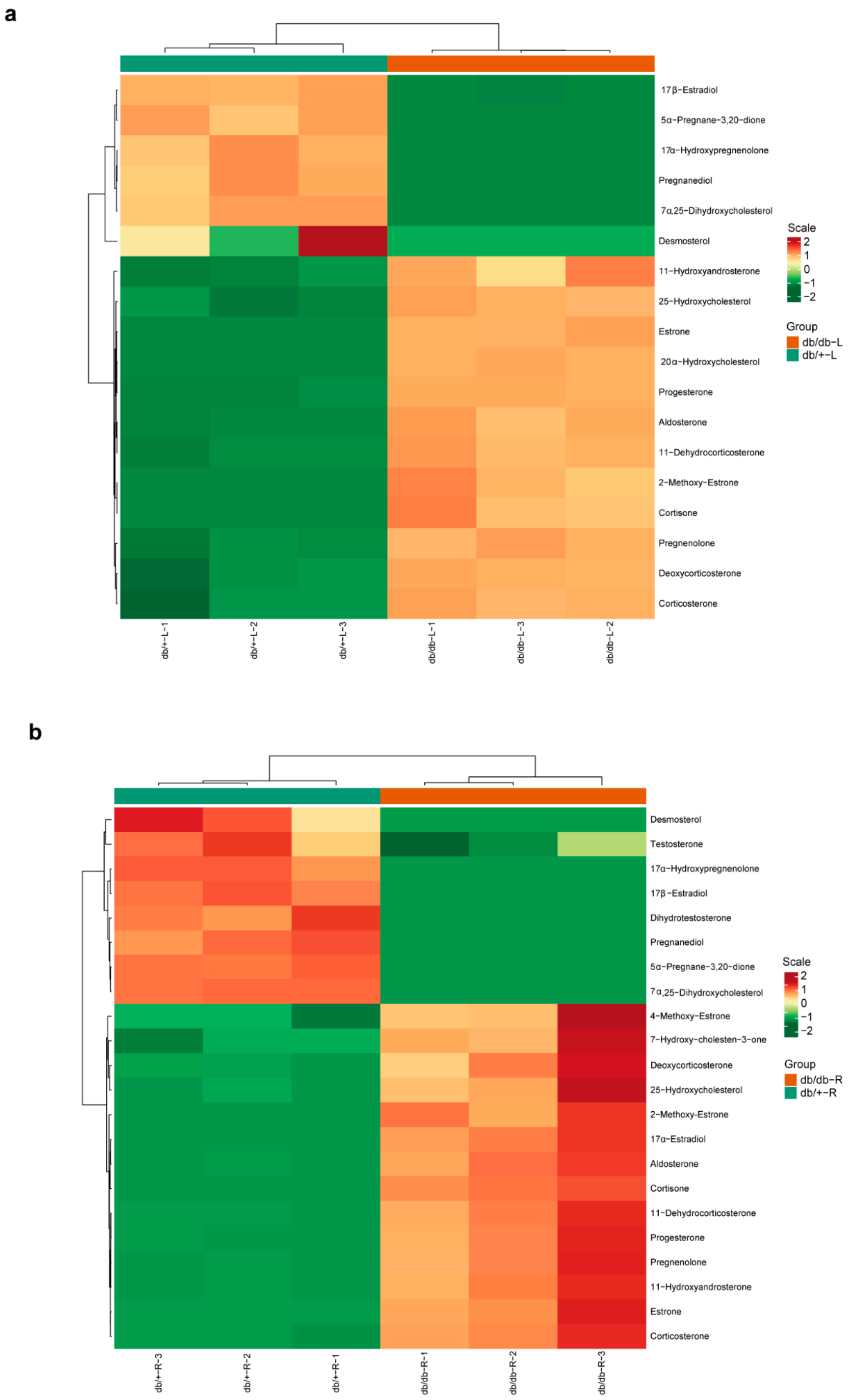

Figure S4. Heatmap of adrenal steroid profile in db/db left vs. db/+ left (**a**) and db/db right vs. db/+ right (**b**).

Supplementary Table

Table S1 Primers used in RT-qPCR experiments

Gene Symbol	NCBI Accession No.	Product Size	Primer sequence (5' to 3')
Hsd3b1	NM_001304800.1	85	TGCTGCACAGCCCTCCTA<br>TCCATCCAGCCATGGTCAAC
Cyp11b2	NM_009991.4	108	TGGCATTGTGGCGGAACTAA<br>AAGGGGATTGCTGTCGTGTC
Cyp11b1	NM_001033229.3	162	CGCTGCAAATCCTCAGAAGG<br>ACATTGAGGACTGTCCCAGCA
Cyp11a1	NM_001346787.1	189	CCCGGAGAGCTTGTGCAAAT<br>CCCATGCTGAGCCAGATGTC
Cyp21a1	NM_009995.2	160	CTTGGGGATGCAAGATGTGG<br>GTGGGCCTTCCACATGAGAG
Star	NM_011485.5	184	TCGCTACGTTCAAGCTGTGT<br>GCTTCCAGTTGAGAACCAAGC
Acsl1	NM_144823.4	185	GTGGTACAACAGGGAACCCC<br>TCCGTGGCAGTAGACAACAG
Rspo3	NM_028351.3	73	GACAGTTGCCCAGAAGGGTT<br>TGGCCTCACAGTGTACAATACT
Abcb1b	NM_011075.2	145	AGTGGCTCTTGAAGCCGTAA<br>AAACTCCATCACCACCTCACG
Shh	NM_009170.3	168	ACGTAGCCGAGAAGACCCTA<br>ACTTGTCTTTGCACCTCTGAGT
Bmp4	NM_007554.3	141	GAGCCATTCCGTAGTGCCAT<br>AGGAATCATGGTGTCTTGACAGA
Ptch1	NM_008957.3	154	GCAGATTTCCAAGGGGAAGGC<br>CACAGCGAAGGCCCCAAATA
Actb	NM_007393.5	89	CACTGTCGAGTCGCGTCC<br>TCATCCATGGCGAACTGGTG
